# Antipsychiatry: Meeting the challenge

**DOI:** 10.4103/0019-5545.31627

**Published:** 2006

**Authors:** Amit Ranjan Basu

**Affiliations:** Independent Researcher, BE 318, Salt Lake, Kolkata 700064, West Bengal e-mail: amitrbasu53@gmail.com

Sir,

The editorial ‘Antipsychiatry: Meeting the challenge’^1^ is interesting and thought-provoking. When the discipline of psychiatry was being impacted by the *Antipsychiatry* movement in the 1970s, there were no comments or even any critique on this topic in the *Indian Journal of Psychiatry!* It is good to see that our journal, albeit after 35 years, has discussed the issue editorially in the present context; it has also tried to strike a balance between the two opposing views: ‘…the willingness to accept the valid criticisms and the possible abuses or “wrongs” of psychiatry, as well as to be open to a dialogue or multilogue and review of practices if required would not only be strategically sound but also ethically warranted.’

This has opened up possibilities to practice psychiatry not determined by biology alone but a multiverse of episteme that contributes to our knowledge of mental healing. In a recent article, Paul Grobstein, a neuroscientist, has seen science as a culture of ‘story telling and story revising’.^2^

However, unlike Grobstein, who wanted to keep science within the boundaries of positivism, I would like to comment on the issue of *Antipsychiatry* and *Postpsychiatry.* The concept of *Antipsychiatry* has lost ground in the present context because of its analytical category being oppositional, dichotomous and binary. Such analyses will eventually take a linear and progressive course, proposing another modernist utopia. The concept of *Postpsychiatry* moved away from this trap and interpreted contemporary changes using tools of continental philosophy including postmodernism. Thomas and Bracken^3^ have explained the differences between ‘anti’ and ‘critical’ psychiatry and argued why psychiatry needs to be analysed from pluralistic viewpoints by challenging the omnipotence and centrality of a deterministic and positivist position. Similar issues were raised 4 years ago by a scholar from the University of Pittsburgh, USA.^4^

Our situation is different from Bracken and Bradley because our modernity is received through colonialism and can best be called postcolonial when the debate on de-colonization is quite vibrant.^5^ For us, psychiatry brings the challenge of negotiating both modern and non-modern discourses and constructing a course of development that acknowledges the multiverse we live in. This may help in generating *our* own theories of psychiatry.
